# Cerebral Hyperperfusion after Revascularization Inhibits Development of Cerebral Ischemic Lesions Due to Artery-to-Artery Emboli during Carotid Exposure in Endarterectomy for Patients with Preoperative Cerebral Hemodynamic Insufficiency: Revisiting the “Impaired Clearance of Emboli” Concept

**DOI:** 10.3390/ijms17081261

**Published:** 2016-08-03

**Authors:** Kentaro Fujimoto, Yoshiyasu Matsumoto, Kohki Oikawa, Jun-ichi Nomura, Yasuyoshi Shimada, Shunrou Fujiwara, Kazunori Terasaki, Masakazu Kobayashi, Kenji Yoshida, Kuniaki Ogasawara

**Affiliations:** 1 Department of Neurosurgery, School of Medicine, Iwate Medical University, 19-1 Uchmaru, 020-8505 Morioka, Japan; norimori@iwate-med.ac.jp (K.F.); yoshiyasu.matumoto@gmail.com (Y.M.); hchrt770@yahoo.co.jp (K.O.); pbx1vfuj@yahoo.co.jp (J.-i.N.); khata@iwate-med.ac.jp (Y.S.); shunfuji@iwate-med.ac.jp (S.F.); kobamasa@iwate-med.ac.jp (M.K.); kenyoshi@iwate-med.ac.jp (K.Y.); 2 Cyclotron Research Center, School of Medicine, Iwate Medical University, 19-1 Uchmaru, 020-8505 Morioka, Japan; ktera@iwate-med.ac.jp

**Keywords:** artery-to-artery embolism, carotid endarterectomy, cerebral hemodynamic insufficiency, cerebral hyperperfusion, ischemic lesion

## Abstract

The purpose of the present study was to determine whether cerebral hyperperfusion after revascularization inhibits development of cerebral ischemic lesions due to artery-to-artery emboli during exposure of the carotid arteries in carotid endarterectomy (CEA). In patients undergoing CEA for internal carotid artery stenosis (≥70%), cerebral blood flow (CBF) was measured using single-photon emission computed tomography (SPECT) before and immediately after CEA. Microembolic signals (MES) were identified using transcranial Doppler during carotid exposure. Diffusion-weighted magnetic resonance imaging (DWI) was performed within 24 h after surgery. Of 32 patients with a combination of reduced cerebrovascular reactivity to acetazolamide on preoperative brain perfusion SPECT and MES during carotid exposure, 14 (44%) showed cerebral hyperperfusion (defined as postoperative CBF increase ≥100% compared with preoperative values), and 16 (50%) developed DWI-characterized postoperative cerebral ischemic lesions. Postoperative cerebral hyperperfusion was significantly associated with the absence of DWI-characterized postoperative cerebral ischemic lesions (95% confidence interval, 0.001–0.179; *p* = 0.0009). These data suggest that cerebral hyperperfusion after revascularization inhibits development of cerebral ischemic lesions due to artery-to-artery emboli during carotid exposure in CEA, supporting the “impaired clearance of emboli” concept. Blood pressure elevation following carotid declamping would be effective when embolism not accompanied by cerebral hyperperfusion occurs during CEA.

## 1. Introduction

It has been previously reported that hemodynamic and embolic mechanisms are closely linked, and they may act together to cause cerebral ischemia [1,2]. The authors suggested that clearance of emboli from a proximal lesion may be decreased by low blood flow velocity in a cerebral artery, which may lead to ischemia from emboli in poorly perfused areas of the brain. In support of this, several previous studies showed that there was a relationship between embolic and hemodynamic mechanisms, particularly in border zone regions having impaired wash-out due to artery-to-artery embolism in cases of middle cerebral artery stenosis [3,4] or in new postoperative neurological deficits caused by emboli developing during carotid artery stenting associated with intraprocedural lower middle cerebral artery blood flow velocity [5].

More than 70% of the intraoperative procedure-related strokes that occur during carotid endarterectomy (CEA) are due to surgical site embolisms [6]. When intraoperative transcranial Doppler (TCD) is used to monitor the middle cerebral artery (MCA), more than 90% of patients undergoing CEA are found to have microembolic signals (MES) [6,7,8]. However, the stage of CEA determines the quality and quantity of MES that are detected [6,8,9]. When the carotid arteries are being exposed, plaque that has not been removed is exposed to blood flow and can be a source of emboli. In such cases, emboli can be dislodged from the surgical site into the intracranial arteries during manipulation of the carotid arteries [8]. In addition, the MES that are detected are considered to represent solid masses, because the target vessel is closed while the vessel is being exposed [10]. However, once the carotid artery walls are incised for the endarterectomy, many harmless gaseous MES may be seen during carotid declamping as air enters the arterial lumina [8,11]. MES detection while the artery is being exposed has been shown to be correlated with the development of post-CEA cerebral ischemic lesions on diffusion-weighted imaging (DWI) [7,8,9,11,12,13]. Furthermore, these ischemic lesions that are related to the emboli generated during carotid artery exposure have been shown to be related to preoperative hemodynamic cerebral compromise, such as reduced cerebrovascular reactivity (CVR) to acetazolamide [14], providing support for the concept of “impaired clearance of emboli”.

Cerebral hyperperfusion is defined as a major increase in ipsilateral cerebral blood flow (CBF) after surgical repair of carotid stenosis that is well above the metabolic demands of the brain tissue; it is another adverse event following CEA [15]. It occasionally evolves into cerebral hyperperfusion syndrome, whose characteristic manifestations include face and eye pain, unilateral headache, seizure, focal neurological symptoms, and disturbance of consciousness secondary to intracerebral hemorrhage or cerebral edema [15,16,17,18]. Impairment of the cerebral hemodynamic reserve before surgery may be related to post-CEA hyperperfusion, and quick normalization of perfusion pressure after CEA may produce hyperperfusion in brain regions with diminished autoregulation from chronic ischemia [17,18]. This hypothesis is consistent with the observation that reduced CVR to acetazolamide prior to surgery is a significant predictor of hyperperfusion after CEA [19,20,21].

Thus, both cerebral ischemic lesions due to artery-to-artery embolism and cerebral hyperperfusion may develop simultaneously during CEA in patients with preoperative cerebral hemodynamic impairment. When broadening the interpretation of the “impaired clearance of emboli” concept, blood flow greater than the normal level can inhibit development of ischemic lesions due to emboli in the brain, and research regarding the influence of cerebral hyperperfusion on the development of cerebral ischemic lesions due to artery-to-artery embolism seems interesting from the standpoint of this concept.

The aim of the present study was to determine whether broadening the interpretation of the “impaired clearance of emboli” concept is correct, namely cerebral hyperperfusion after revascularization inhibits development of cerebral ischemic lesions due to intraoperative artery-to-artery emboli. In order to do this, the relationship between development of DWI-characterized postoperative cerebral ischemic lesions and cerebral hyperperfusion was investigated in patients with a combination of preoperatively reduced CVR to acetazolamide on brain perfusion single-photon emission computed tomography (SPECT) and MES on TCD during carotid artery exposure in CEA.

## 2. Results

### 2.1. Trial Profile

**Figure 1:**
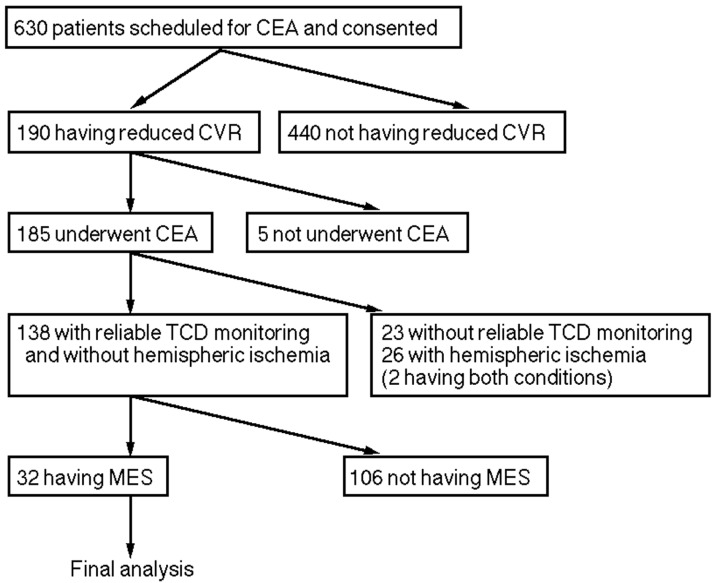
Trial profile showing the flow chart of patient numbers from initial screening to final analysis. Patients who did not have preoperative reduced cerebrovascular reactivity (CVR), did not undergo carotid endarterectomy (CEA), did not have reliable intraoperative transcranial Doppler (TCD) monitoring, had hemispheric ischemia during carotid clamping, and did not have microembolic signals (MES) during carotid exposure were excluded from the study.

Figure 1 shows the patient flow chart for this study. Six hundred and thirty patients with ipsilateral internal carotid artery (ICA) stenosis ≥70% and useful residual function were scheduled for CEA and consented to participate in the present study. Of these 630 patients, 190 were defined as having reduced CVR to acetazolamide. Of these 190 patients, five did not undergo CEA and were excluded from the analysis. Of the 185 patients who underwent CEA, 23 did not show reliable TCD monitoring during carotid exposure because of failure to obtain an adequate bone window, and 26 showed electroencephalography (EEG)-defined hemispheric ischemia during ICA clamping; these 47 patients (two had both conditions) were excluded from the analysis. Of the remaining 138 patients, 32 had MES during exposure of the carotid arteries and were finally analyzed. Data acquisition with brain perfusion SPECT was completed within 3 h after declamping of the ICA in all these 32 patients. They all underwent DWI 24 h after surgery.

### 2.2. Clinical Characteristics

The mean age of the 32 patients (29 men, three women) was 71.7 ± 4.5 (mean ± standard deviation (SD)) years (range of 63–85 years). Twenty-six patients had preoperative hypertension, and 23 patients received antihypertensive drugs (calcium antagonist alone for five, angiotensin receptor blocker alone for 13 and both for five). Thirteen patients had preoperative diabetes mellitus, and all these patients received antidiabetic drugs. Fifteen patients had preoperative dyslipidemia, and 12 patients received a statin (strong statin for six). Seven patients had ischemic heart or valvular disease that did not satisfy the criteria for high-risk factors for CEA in the Stenting and Angioplasty with Protection in Patients at High Risk for Endarterectomy (SAPPHIRE) study (congestive heart failure, abnormal stress test, or need for open-heart surgery) [22]. None of the 32 patients had atrial fibrillation. Twenty-five patients had ipsilateral carotid territory symptoms, and seven patients had asymptomatic ICA stenosis. The overall average degree of ICA stenosis was 85.4% ± 8.8% (range, 70%–99%), with nine patients showing >70% stenosis or occlusion in the contralateral ICA. Preoperative CBF and CVR to acetazolamide were 33.1 ± 6.1 mL/100 g/min (range of 22.6–44.5 mL/100 g/min) and 8.6% ± 6.9% (range of −8.3%–18.0%), respectively. Preoperative systolic blood pressure was 133.6 ± 11.7 mmHg (range of 111–156 mmHg). The number of MES ranged from one to 14 (4.3 ± 3.9). The interval from the first MES to ICA declamping ranged from 34 to 59 min (45.3 ± 6.4 min). The interval from the last MES to ICA declamping ranged from 35 to 65 min (46.8 ± 6.6 min). Mean systolic blood pressure during carotid exposure was 113.8 ± 10.7 mmHg (range of 95–138 mmHg). Mean duration of ICA clamping was 36.0 ± 5.7 min (range of 28–47 min). Mean systolic blood pressure in the post-carotid declamping period in surgery was 121.4 ± 11.9 mmHg (range of 105–141 mmHg). Mean systolic blood pressure in the postoperative period (within 24 h after surgery) was 127.8 ± 12.6 mmHg (range of 109–147 mmHg). The mean rate of blood pressure–measured points with successfully controlled blood pressure to all measured points in the post-carotid declamping period in surgery was 85.9% ± 3.9% (range of 72%–93%). The mean rate of blood pressure–measured points with successfully controlled blood pressure to all the measured points in the postoperative period was 76.1% ± 4.9% (range of 67%–92%).

### 2.3. Postoperative Events

In the 32 patients studied, postoperative CBF was 52.9 ± 17.5 mL/100 g/min (range of 35.8–90.5 mL/100 g/min); 14 patients (44%) met CBF criteria for cerebral hyperperfusion. Sixteen patients (50%) developed new postoperative ischemic lesions on DWI 24 h after surgery in the cortex and/or white matter in the cerebral hemisphere ipsilateral to CEA. All new ischemic lesions were spotty, and their diameters were 1 cm or less. Five (16%) of 32 patients studied developed new neurological deficits after recovery from general anesthesia. All deficits included slight hemiparesis contralateral to the CEA. These deficits resolved completely within 12 h in these five patients, and they underwent additional DWI between 6 and 8 h after surgery and had new postoperative ischemic lesions on both the first (6 to 8 h after surgery) and second (24 h after surgery) postoperative DWI examinations.

### 2.4. Postoperative Cerebral Hyperperfusion vs. Diffusion-Weighted Imaging (DWI)-Characterized Postoperative Cerebral Ischemic Lesions

Results of univariate analyses of factors related to the development of DWI-characterized postoperative cerebral ischemic lesions are shown in Table 1. The postoperative CBF and the incidence of postoperative cerebral hyperperfusion were significantly higher in patients without than in those with DWI-characterized postoperative cerebral ischemic lesions. Other variables were not significantly associated with DWI-characterized postoperative cerebral ischemic lesions. After eliminating variables that were closely related, the following items with values of *p* < 0.2 in univariate analyses were adopted as confounders in the logistic regression model for multivariate analysis: degree of ICA stenosis and postoperative CBF or postoperative cerebral hyperperfusion (since the latter two interacted, each item was adopted individually). This analysis showed that greater postoperative CBF (95% confidence interval, 0.616–0.938; *p* = 0.0104) or postoperative cerebral hyperperfusion (95% confidence interval, 0.001–0.179; *p* = 0.0009) was significantly associated with the absence of DWI-characterized postoperative cerebral ischemic lesions.

**Figure 2:**
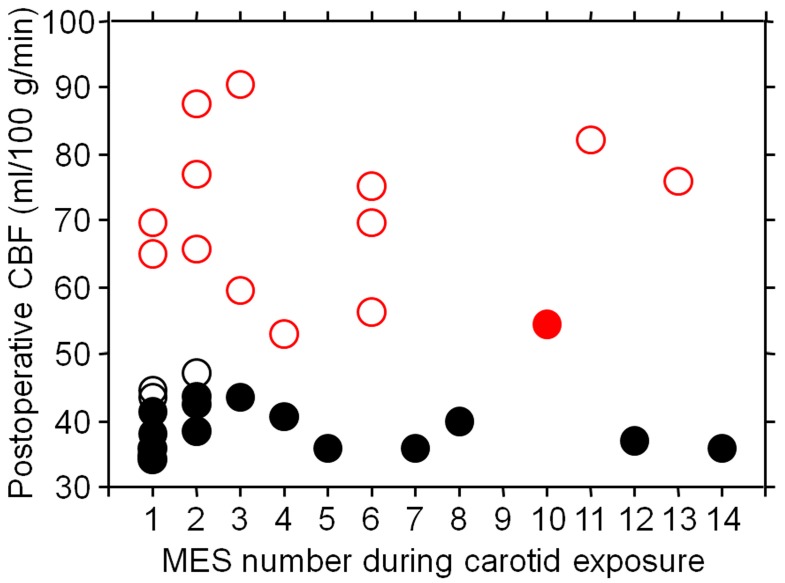
Relationships between the number of microembolic signals (MES), postoperative CBF (cerebral blood flow), cerebral hyperperfusion, and the development of diffusion-weighted imaging (DWI)-characterized postoperative cerebral ischemic lesions. Closed and open circles indicate patients with and without DWI-characterized postoperative cerebral ischemic lesions, respectively. Red and black circles indicate patients with and without postoperative cerebral hyperperfusion (defined as postoperative CBF increase ≥100% compared with preoperative values), respectively. Whereas 15 (83%) of 18 patients without postoperative cerebral hyperperfusion showed DWI-characterized postoperative cerebral ischemic lesions, only one (7%) of 14 patients with hyperperfusion had these ischemic lesions.

Figure 2 shows the relationships between the number of MES, postoperative CBF, cerebral hyperperfusion, and the development of DWI-characterized postoperative cerebral ischemic lesions. Postoperative CBF in patients with cerebral hyperperfusion ranged from mean + 3.9 SD to mean + 11.8 SD of the control value. Whereas 15 (83%) of 18 patients without postoperative cerebral hyperperfusion showed DWI-characterized postoperative cerebral ischemic lesions, only one (7%) of 14 patients with hyperperfusion had these ischemic lesions.

### 2.5. Case Presentation

**Figure 3:**
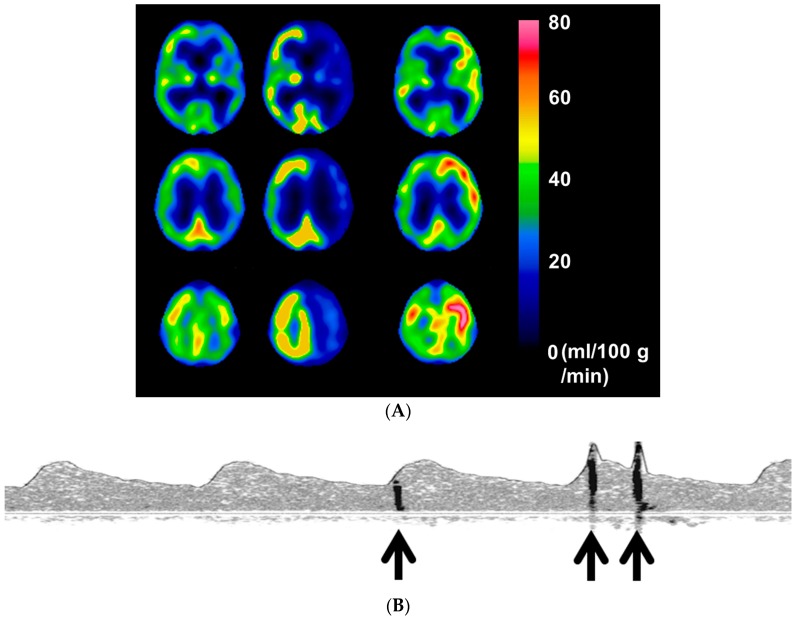

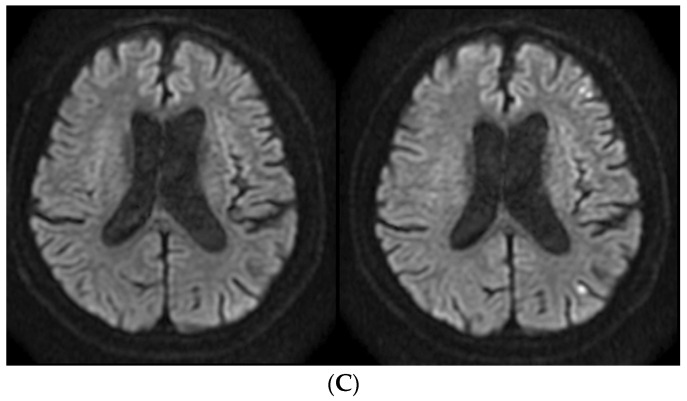
(**A**) Preoperative brain perfusion single-photon emission computed tomography in a 74-year-old man with symptomatic left internal carotid artery stenosis (90%) shows reduced cerebral blood flow (**left**) and reduced cerebrovascular reactivity to acetazolamide (**center**) in the left cerebral hemisphere where hyperperfusion develops immediately after surgery (**right**); (**B**) Transcranial Doppler recording during exposure of the carotid arteries in the patient of Figure 3A shows three microembolic signals (**arrows**) in the power spectrum display of left middle cerebral artery blood flow. This patient had a total of 10 microembolic signals during exposure of the carotid arteries; (**C**) A diffusion-weighted image 6 h after surgery in the patient of Figure 3A,B shows development of new postoperative multiple high-intensity lesions in the left cerebral hemisphere (**right**) when compared with a preoperative image (**left**). These lesions did not change on diffusion-weighted imaging 24 h after surgery. This patient suffered slight motor weakness in the right upper extremity after recovery from general anesthesia, and this deficit resolved completely within 12 h.

Figure 3 shows images of brain perfusion SPECT, TCD, and DWI in a 74-year-old man with symptomatic ICA stenosis (90%) showing DWI-characterized postoperative cerebral ischemic lesions due to MES during exposure of the carotid arteries despite development of cerebral hyperperfusion after left CEA.

## 3. Discussion

### 3.1. Findings

The present study demonstrated that cerebral hyperperfusion after revascularization inhibits the development of cerebral ischemic lesions due to artery-to-artery emboli during carotid exposure in CEA for patients with preoperatively impaired cerebral hemodynamics, supporting the “impaired clearance of emboli” concept when broadening its interpretation.

### 3.2. Reason of Patient Exclusion

Hemodynamic cerebral ischemia due to hemispheric cerebral hypoperfusion during ICA clamping, as well as emboli from the surgical site, plays a significant role in the development of new ischemic lesions after CEA [11,23]. Intraoperative EEG monitoring is the most widely used and best documented method for the detection of hemispheric cerebral hypoperfusion due to carotid clamping [24]. To investigate the development of cerebral ischemic lesions caused by MES rather than by hemispheric cerebral hypoperfusion during ICA clamping, patients with EEG-defined hemispheric ischemia during ICA clamping were excluded from the present study.

### 3.3. Data Interpretation

In the present study, 44% of patients with preoperatively reduced CVR to acetazolamide showed cerebral hyperperfusion immediately after surgery. This incidence was comparable to a previous study [19,20,21]. All postoperative ischemic lesions on DWI that were newly developed in the cortex and/or white matter in the cerebral hemisphere ipsilateral to CEA were spotty, and their diameters were 1 cm or less. Furthermore, the duration of ICA clamping did not differ between patients with and without DWI-characterized postoperative cerebral ischemic lesions. Thus, these ischemic lesions were possibly due to artery-to-artery embolism rather than cerebral hemispheric ischemia during ICA clamping. More than 80% of patients with a combination of preoperatively reduced CVR to acetazolamide and MES during carotid exposure, when they did not exhibit postoperative cerebral hyperperfusion, developed DWI-characterized postoperative cerebral ischemic lesions, which corresponded with previous findings [14].

In the present study, preoperative CBF, preoperative CVR to acetazolamide, mean systolic blood pressure during carotid exposure, and interval from the first or last MES to ICA declamping did not differ between patients with and without DWI-characterized postoperative cerebral ischemic lesions. Thus, perfusion in the cerebral hemisphere ipsilateral to surgery during carotid exposure and the duration of cerebral ischemia caused by emboli until ICA declamping might be equivalent between these two subgroups of patients. The duration of ICA clamping also did not differ between them. Nevertheless, greater postoperative CBF or postoperative cerebral hyperperfusion (postoperative CBF ≥ mean + 3.9 SD of the control value) was associated with the absence of DWI-characterized postoperative cerebral ischemic lesions, and only 7% of patients with a combination of MES during carotid exposure and postoperative cerebral hyperperfusion developed these lesions. These findings suggested that blood flow that increased far beyond the normal level might clear cerebral emboli generated from the surgical site, inhibiting the development of ischemic lesions. These support the “impaired clearance of emboli” concept if the interpretation of this concept is broadened.

We have another hypothesis regarding the correlation between postoperative cerebral hyperperfusion and the development of ischemic lesions by emboli in patients with reduced CVR to acetazolamide. Reduced CVR to acetazolamide implies a chronic reduction in cerebral perfusion pressure and poor collateral blood flow [25,26,27]. When emboli generated from a lesion in the ICA acutely disturb blood flow in the cerebral artery, cerebral blood flow may be further decreased in the affected vascular territory with the pre-existing chronic reduction in cerebral perfusion pressure. However, if cerebral ischemic lesions have not yet formed between the onset of emboli and ICA declamping (the interval ranged from approximately 30 min to 60 min in the present study), hyperperfusion after ICA declamping may lead to an extreme increase in collateral blood flow to the affected vascular territory, inhibiting the postoperative development of new cerebral ischemic lesions.

### 3.4. Future Directions

The present study suggests that CBF greater than the normal level after declamping of the ICA can inhibit development of cerebral ischemic lesions due to emboli from the surgical site during exposure of the carotid arteries. On the other hand, postoperative cerebral hyperperfusion, which is defined as a postoperative CBF increase of ≥100% when compared to preoperative values, occasionally evolves into cerebral hyperperfusion syndrome, leading to intracerebral hemorrhage [15,16,17,18]. Strict postoperative control of blood pressure (systolic blood pressure < 90 mmHg) reportedly prevents the development of intracerebral hemorrhage [16,20]. Further, intraoperative monitoring of MCA flow velocity using TCD or regional cerebral oxygen saturation using near-infrared spectroscopy are reliable methods of identifying patients with cerebral hyperperfusion following declamping of the ICA during CEA [28,29]. On the basis of these findings, we propose a practical clinical algorithm to prevent development of embolic ischemic events and hyperperfusion-related hemorrhage in CEA: when the intraoperative monitoring suggests development of cerebral hyperperfusion following declamping of the ICA, the blood pressure should then be reduced; when the intraoperative monitoring suggests development of embolism from the surgical site during carotid exposure that is not accompanied by cerebral hyperperfusion, the blood pressure should be elevated above the preoperative value following declamping of the ICA. Further investigation to determine whether the latter procedure prevents development of cerebral ischemic lesions would be of benefit, although, in the present study, blood pressure was reduced for all patients regardless of the presence or absence of cerebral hyperperfusion after declamping of the ICA.

### 3.5. Study Limitations

Although TCD detects emboli generated from the surgical site of the carotid arteries as MES, it cannot provide information about the size and characteristics of each embolus, which may affect the development of postoperative cerebral ischemic lesions. The present results did not take into account these two factors.

## 4. Materials and Methods

### 4.1. Subjects

The present study was designed as a prospective, observational study. This study was approved by the Regional Ethical Board in Iwate Medical University (H22-3) and was in compliance with the Helsinki Declaration, and written, informed consent was obtained from all patients or their next of kin prior to participation.

Of symptomatic or asymptomatic patients with ipsilateral ICA stenosis ≥70%, as per the North American Symptomatic Carotid Endarterectomy Trial [30], on angiography/arterial catheterization, and useful residual function (modified Rankin scale score 0, 1, or 2) who were scheduled for CEA of the carotid bifurcation, those who satisfied the following inclusion criteria were prospectively selected for the present study: having preoperatively reduced CVR to acetazolamide according to the methods described below (see “*4.2.* *CBF Measurements*” section); undergoing CEA; and having MES during exposure of the carotid arteries under reliable TCD monitoring according to the methods described below (see “*4.3.* *TCD Monitoring*” section). Patients who showed electroencephalography (EEG)-defined cerebral hemispheric ischemia during ICA clamping according to the methods described below (see “*4.5. Preoperative, Intraoperative, and Postoperative Management*” section) were excluded from the present study.

### 4.2. CBF Measurements

CBF was assessed using [^123^I]N-isopropyl-*p*-iodoamphetamine (IMP) and SPECT with a ring-type scanner (Headtome-SET 080; Shimadzu, Kyoto, Japan) within 14 days before and immediately after CEA. CBF measurement with acetazolamide challenge was also performed before CEA. The [^123^I]IMP SPECT study with and without acetazolamide challenge was performed as described previously [31,32]. After a 1 min intravenous infusion of 222 MBq of [^123^I]IMP (5 mL volume) at a constant rate of 5 mL/min and a 1 min infusion of physiologic saline at the same rate, data acquisition was performed at a midscan time of 30 min after the [^123^I]IMP administration for a scan duration of 20 min. At 10 min after the beginning of the [^123^I]IMP infusion, arterial blood (1 mL) was taken from the brachial artery. The whole-blood radioactivity of each blood sample obtained was measured using a well counter that was cross-calibrated to the SPECT scanner. All reconstructed SPECT images were corrected for the radioactive decay of ^123^I back to the [^123^I]IMP injection start time, normalized by the data collection time and cross-calibrated to the well counter system. The CBF images were calculated according to the [^123^I]IMP-autoradiography method [31,32]. The whole-blood radioactivity counts of the single blood sample were referred to the standard input function.

**Figure 4:**
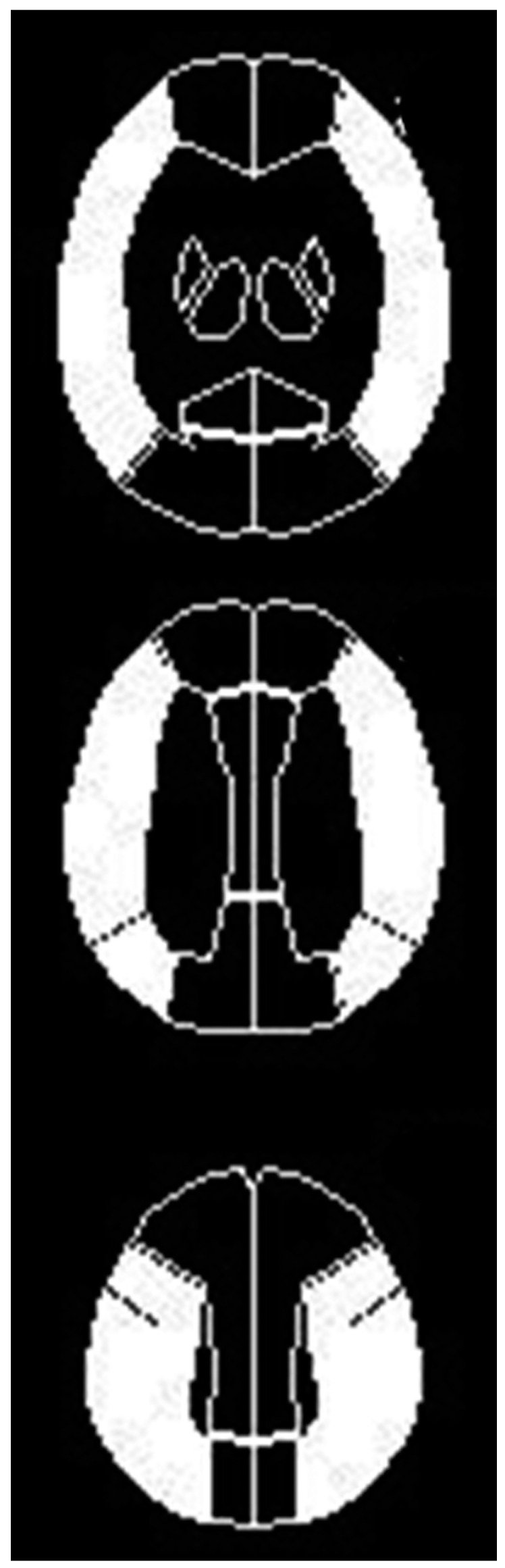
Diagrams show the regions of interests (ROIs) for a three-dimensional, stereotactic ROI template to automatically place constant ROIs on brain perfusion single-photon emission computed tomography images. White ROIs indicate middle cerebral artery territories (precentral, central, parietal, angular, and temporal).

All SPECT images were transformed into standard brain size and shape by linear and nonlinear transformations using statistical parametric mapping 2 software for anatomical standardization [33]. A three-dimensional stereotactic region-of-interest (ROI) template was used to automatically place 318 constant ROIs in both cerebral and cerebellar hemispheres [34]. ROIs were grouped into 10 segments (callosomarginal, pericallosal, precentral, central, parietal, angular, temporal, posterior, hippocampal, and cerebellar) in each hemisphere according to the arterial supply. Five (precentral, central, parietal, angular, and temporal) of these 10 segments were combined and defined as an ROI perfused by the middle cerebral artery (MCA) (Figure 4).

The mean value of all pixels in the MCA ROI in the cerebral hemisphere ipsilateral to CEA was calculated. Preoperative CVR to acetazolamide in the cerebral hemisphere ipsilateral to CEA was calculated as follows: CVR (%) = [(CBF with acetazolamide challenge − CBF at the resting state)/CBF at the resting state] × 100. For CBF in the resting state and CVR to acetazolamide, data described previously ((mean ± SD), 35.9 ± 4.4 mL/100 g/min and 36.8% ± 9.2%, respectively) were used as control values, and decreased CVR to acetazolamide was defined as less than mean − 2 SD of the control value (18.4%) [31]. In each patient, cerebral hyperperfusion was defined as a postoperative CBF increase of ≥100% (i.e., a doubling) when compared to preoperative values in the MCA ROI ipsilateral to the side of surgery [20].

### 4.3. Transcranial Doppler (TCD) Monitoring

TCD was performed using a PIONEER TC2020 system (EME, Uberlingen, Germany; software version 2.50, 2 MHz probe; diameter, 1.5 cm; insonation depth, 40–66 mm; scale, −100 and +150 cm/s; sample volume, 2 mm; 64 point fast Fourier transform; fast Fourier transform length, 2 mm; fast Fourier transform overlap, 60%; high-pass filter, 100 Hz; detection threshold, 9 dB; minimum increase time, 10 ms) for insonation of the MCA ipsilateral to the carotid artery undergoing CEA. TCD data were stored on a hard disk using a coding system and later analyzed manually by a clinical neurophysiologist who was blinded to patient information. MES were identified during exposure of the carotid arteries (from skin incision to ICA clamping) according to the recommended guidelines [35].

### 4.4. Magnetic Resonance Imaging

DWI was performed using a 1.5 T whole-body imaging system (Signa MR/I; GE Healthcare, Milwaukee, WI, USA) within three days before and 24 h after surgery.

A neuroradiologist who was blinded to patient clinical information analyzed the images and determined whether new ischemic lesions had developed postoperatively.

### 4.5. Preoperative, Intraoperative, and Postoperative Management

Blood pressure was measured at the upper arm using an automatic sphygmomanometer with the oscillometric method, and mean systolic blood pressure in the morning for the three days before surgery was defined as the preoperative value for each patient. Patients received medications including antihypertensive and antidiabetic drugs and statins until the evening of the day before CEA was performed. All patients received a single antiplatelet drug until the morning of the day on which CEA was performed. For all patients, surgery was conducted under general anesthesia, which was induced with etomidate/fentanyl and maintained with O_2_/propofol. A bolus of heparin (5000 international units) was given prior to ICA clamping. Blood pressure was measured in the same fashion as preoperatively every 5 min throughout surgery. The EEG was recorded, and a clinical neurophysiologist monitored the recordings continuously during the surgical procedure. The presence of unilateral or bilateral decreases of alpha and beta activity during ICA clamping, with or without simultaneous increases of theta or delta activity, was defined as development of cerebral hemispheric ischemia by the clinical neurophysiologist [24]. In this situation, an intraluminal shunt was introduced. From declamping of the ICA to the third postoperative day, attempts were made to reduce systolic blood pressure to below 90% of the preoperative value using intravenous injection of the calcium antagonist nicardipine. Blood pressure was measured in the same fashion as preoperatively every 1 h until 24 h after surgery. When systolic blood pressure was <90% of the preoperative value at a blood pressure-measured point, the point was defined as having successfully controlled blood pressure. Patients received the same drugs as preoperative medications from the second postoperative day.

### 4.6. Statistical Analysis

Data are expressed as means ± SD. The relationship between each variable and DWI-characterized postoperative cerebral ischemic lesions was evaluated by univariate analysis using the Mann-Whitney U test or the χ^2^ test. Hypertension was defined as preoperative systolic blood pressure ≥140 mmHg, preoperative diastolic blood pressure ≥90 mmHg or preoperatively receiving antihypertensive drugs; diabetes mellitus was defined as preoperative hemoglobin A1c ≥6.5% or preoperatively receiving antidiabetic drugs; dyslipidemia was defined as preoperative plasma low density lipoprotein (LDL) cholesterol ≥140 mg/dL, preoperative plasma high density lipoprotein (HDL) cholesterol <40 mg/dL, preoperative plasma triglyceride ≥150 mg/dL, or preoperatively receiving statins. Multivariate statistical analysis of factors related to DWI-characterized postoperative cerebral ischemic lesions was also performed using a logistic regression model. Variables with *p* < 0.2 on univariate analyses were selected for analysis in the final model. Differences were deemed significant for values of *p* < 0.05.

## 5. Conclusions

The present study demonstrated that cerebral hyperperfusion after revascularization inhibits the development of cerebral ischemic lesions due to artery-to-artery emboli during carotid exposure in CEA for patients with preoperatively impaired cerebral hemodynamics, supporting the “impaired clearance of emboli” concept when its interpretation is broadened.

## Figures and Tables

**Table 1:** Univariate analysis of factors related to development of diffusion-weighted imaging (DWI)-characterized postoperative cerebral ischemic lesions.

Variable	DWI-Characterized Ischemic Lesions		*p*
	Yes	No	
	(*n* = 16)	(*n* = 16)	
Age (years, mean ± SD)	72.8 ± 5.4	70.7 ± 3.2	0.2557
Male sex	15 (94%)	14 (88%)	>0.9999
Hypertension	12 (75%)	14 (88%)	0.6539
Preoperative antihypertensive drugs	10 (63%)	13 (81%)	0.4331
Preoperative calcium antagonist	5 (31%)	5 (31%)	>0.9999
Preoperative angiotensin receptor blocker	8 (50%)	10 (63%)	0.7224
Diabetes mellitus	6 (38%)	7 (44%)	>0.9999
Preoperative antidiabetic drugs	6 (38%)	7 (44%)	>0.9999
Dyslipidemia	7 (44%)	8 (50%)	>0.9999
Preoperative statins	5 (31%)	7 (44%)	0.7160
Preoperative strong statins *	2 (12%)	4 (25%)	0.6539
Preoperative aspirin	6 (38%)	4 (25%)	0.7043
Preoperative clopidogrel	10 (63%)	12 (75%)	0.7043
Ischemic heart or valvular disease	3 (19%)	4 (25%)	>0.9999
Symptomatic lesion	14 (88%)	11 (69%)	0.3944
Degree of ICA stenosis (%, mean ± SD)	83.1 ± 9.0	87.7 ± 8.2	0.1258
Bilateral lesions	4 (25%)	5 (31%)	>0.9999
Preoperative CBF (mL/100 g/min, mean ± SD)	31.9 ± 5.8	34.4 ± 6.4	0.2581
Preoperative CVR to acetazolamide (%, mean ± SD)	8.7 ± 5.9	8.6 ± 8.0	0.6783
Preoperative systolic blood pressure (mmHg, mean ± SD)	134.5 ± 15.8	132.5 ± 14.2	0.9254
Number of MES (mean ± SD)	4.6 ± 4.3	4.0 ± 3.6	0.8932
Interval from first MES to ICA declamping (min, mean ± SD)	46.1 ± 6.8	44.4 ± 6.1	0.4848
Interval from last MES to ICA declamping (min, mean ± SD)	47.2 ± 7.9	46.3 ± 5.2	0.8353
Mean systolic blood pressure during carotid exposure (mmHg, mean ± SD)	114.2 ± 14.8	113.4 ± 13.0	0.9849
Duration of ICA clamping (min, mean ± SD)	37.2 ± 5.6	34.8 ± 5.6	0.2191
Mean systolic blood pressure after carotid declamping (mmHg, mean ± SD)	122.2 ± 15.1	120.7 ± 13.1	0.9049
Successfully controlled blood pressure after carotid declamping ** (%, mean ± SD)	84.6 ± 5.3	87.2 ± 6.8	0.8954
Mean systolic blood pressure in postoperative period (mmHg, mean ± SD)	128.8 ± 16.8	127.1 ± 17.0	0.9241
Successfully controlled blood pressure in postoperative period *** (%, mean ± SD)	74.5 ± 6.8	78.3 ± 7.8	0.8037
Postoperative CBF (mL/100 g/min, mean ± SD)	39.6 ± 4.8	66.3 ± 15.0	<0.0001
Cerebral hyperperfusion	1 (6%)	13 (81%)	<0.0001

SD, Standard deviation; ICA, Internal carotid artery; CBF, Cerebral blood flow; CVR, Cerebrovascular reactivity; MES, Microembolic signal; *, Including atorvastatin, pitavastatin, and rosuvastatin; **, Rate of blood pressure–measured points with systolic blood pressure <90% of the preoperative value in the post-carotid declamping period in surgery; ***, Rate of blood pressure–measured points with systolic blood pressure <90% of the preoperative value in the postoperative period (within 24 h after surgery).

## Abbreviations

CEA	Carotid endarterectomy
TCD	Transcranial Doppler
MCA	Middle cerebral artery
MES	Microembolic signals
DWI	Diffusion-weighted imaging
CVR	Cerebrovascular reactivity
CBF	Cerebral blood flow
SPECT	Single-photon emission computed tomography
ICA	Internal carotid artery
EEG	Electroencephalography
IMP	[^123^I]N-isopropyl-*p*-iodoamphetamine
ROI	Region-of-interest
SD	Standard deviation
